# Phase II trial of metformin and paclitaxel for patients with gemcitabine-refractory advanced adenocarcinoma of the pancreas

**DOI:** 10.3332/ecancer.2015.563

**Published:** 2015-08-11

**Authors:** Maria Ignez Braghiroli, Anezka C R de Celis Ferrari, Tulio Eduardo Pfiffer, Alexandra Kichfy Alex, Daniela Nebuloni, Allyne S Carneiro, Fernanda Caparelli, Luiz Senna, Juliana Lobo, Paulo Marcelo Hoff, Rachel P Riechelmann

**Affiliations:** Discipline of Radiology and Oncology, Instituto do Cancer do Estado de São Paulo, Universidade de São Paulo, Brazil, Av Dr Arnaldo 251, 12º andar São Paulo, SP, 01246-000 Brazil

**Keywords:** pancreas adenocarcinoma, metformin

## Abstract

**Background:**

In patients with adenocarcinoma of the pancreas, there are no standard second-line regimens. Many pre-clinical studies have shown that metformin alone or when combined with paclitaxel has antitumour effects on this tumour. We have tested here the combination of paclitaxel and metformin for patients with gemcitabine-refractory pancreatic cancer.

**Methods:**

An uncontrolled phase II trial was carried out based on a two–stage Simon’s design, with metformin and paclitaxel for patients with locally advanced or metastatic pancreatic cancer whose disease had progressed during first line treatment with a gemcitabine-based regimen. The primary endpoint was the disease control rate at eight weeks as per response evaluation criteria in solid tumours (RECIST) 1.1. Patients received paclitaxel 80 mg/m^2^ weekly for three weeks every 28 days and metformin 850 mg p.o. t.i.d. continuously until progression or intolerance state was reached.

**Results:**

Twenty patients were enrolled from July 2011 to January 2014: N = 6 (31.6%) achieved the primary endpoint, with all presenting stable disease. Median overall survival (OS) was 128 days (range 17–697) and the median progression free survival (PFS) was 44 days (range 14–210). Eight patients (40%) presented treatment-related G3-4 toxicities with the most common one being diarrhoea.

**Conclusions:**

Despite the encouraging pre-clinical evidence of the antitumour activity of metformin in adenocarcinoma of the pancreas, the primary endpoint of the disease control rate was not met. Besides, the treatment combination was poorly tolerated and could not be studied further. This study highlights the importance of performing clinical trials to reassure preclinical or observational data.

## Background

Patients with advanced adenocarcinoma of the pancreas generally live less than one year even when treated with more effective regimens such as FOLFIRINOX [1]. The median survival of patients whose disease progresses first-line is in the range of weeks. In this setting, there is no standard second-line therapy with only a few regimens demonstrating modest benefits [2, 3]. Therefore, there is an urgent need to develop new treatment options for patients with advanced pancreatic cancer.

Several preclinical and clinical studies have suggested that metformin has antitumour activity in a number of malignancies [4], although the exact antineoplastic mechanism of action is unknown. Studies have suggested that lowering the insulin levels through activation of AMPK could minimise the mitogenic effects of this hormone on cancer cells [5]. Metformin has also been shown to downregulate mTOR, which is a serine/threonine kinase involved in cell energy generation and survival [6]. Metformin antitumour mechanism is still being speculated, but possible explanations involve AMPK activation with downregulation of specificity protein (Sp) transcription factors [7] and inhibition of the insulin/insulin-like growth factor-I (IGF-I) receptor activation [8], *In vitro* experiments using human pancreatic cancer cells showed that metformin abolished signalling normally induced by insulin through G protein-coupled receptor [9]. Even low doses blocked the stimulation of DNA synthesis and cell growth induced by insulin. Likewise, in xenografted models, metformin decreased the growth of pancreatic cancer cells [9].

There is also some clinical evidence that metformin could have antitumour activity in pancreatic adenocarcinoma (PAC). A meta-analysis of 37 studies with 1,535,636 participants with pancreatic, liver, colorectal, breast, and prostate cancers identified a 46% relative risk reduction in pancreatic cancer incidence among diabetic patients who used metformin [10]. A retrospective study from the University of Texas MD Anderson Cancer Centre showed that diabetic patients with pancreatic cancer and who used metformin had a 38% reduced relative risk of death (Hazard ratio HR = 0.62; 95%: confidence interval CI 0.44–0.87; P = 0.006) and improved median survival (15.2 versus 11.1 months, P = 0.004) when compared with the non-metformin group. [11]. However, the current literature on the effects of metformin in patients with PAC has been restricted to patients with diabetes. In addition, clinical trials assessing whether metformin has true antitumour effects in patients with advanced PAC, independently of diabetes, have not yet been performed.

Based on the clinical and preclinical rationale that metformin has antitumour activity in PAC, we have conducted a phase II trial of this drug combined with paclitaxel. The choice to combine metformin with paclitaxel was based on preclinical data that have demonstrated synergism with this combination [12, 13].

## Methods

We performed a single-arm Simon’s two-stage phase II trial to evaluate the efficacy of metformin combined with weekly paclitaxel in patients with metastatic and/or locally advanced adenocarcinoma of the pancreas whose disease had progressed or were intolerant to first-line gemcitabine. The primary objective was to measure the antitumour activity of the experimental therapy. Secondary objectives were safety and tolerability, OS, and PFS. The primary endpoint was disease control rate (DCR) at eight weeks after treatment initiation. DCR was defined as the proportion of patients who presented either complete or partial response or stable disease by RECIST 1.1 criteria.

Patients were gathered from the Gastrointestinal Oncology Clinics at the Instituto do Câncer do Estado de São Paulo, São Paulo, Brazil, which is one of the largest cancer centres in the country. Eligible patients had histologically confirmed metastatic and/or locally advanced adenocarcinoma of the pancreas, presented radiological progression or were intolerant to first-line gemcitabine-based regimens, had performance status 0–2 by ECOG (Eastern Cooperative Oncology Group) scale, life expectancy of at least ten weeks and adequate organic function. Patients were excluded if they were diabetic and were metformin regular users, had any major surgical procedure or radiation within four weeks prior to study entry, history of symptomatic hypoglycemia, any serious medical or psychiatric illness as per clinical judgment, or with pregnancy/lactancy.

The treatment consisted of paclitaxel 80 mg/m^2^ weekly for three consecutive weeks every 28 days (one cycle) and metformin 850mg t.i.d. continuously. Treatment was administered until clinical or radiological progression (whichever came first), unacceptable toxicity, or consent withdrawal. The dose of metformin was chosen based on the dosage commonly used by diabetic patients.

Dose adjustments were preplanned according to level of toxicity presented by the patient. Expected symptoms related to metformin were diarrhoea, nausea, loss of appetite, and metallic taste. Drug interruption for longer than three weeks because toxicity was the criteria for study withdrawal. Patients with grade 3–4 toxicity had treatment delayed until toxicity was grade 1 or resolved. After grade 3 toxicity, patients were re-started on metformin 850 mg b.i.d., and after grade 4 toxicity the new dose should be 500mg b.i.d.

Computed tomography (CT) scans of the chest, abdomen, and pelvis were performed at baseline and at every eight weeks until progression. Patients who had to be taken off study for reasons other than tumour progression were followed with CT scans until progression. Patients were evaluated by study investigators on days 1 and 15 in the first cycle to monitor tolerability and adherence, and every 28 days thereafter. Blood counts and biochemistry were collected prior to every cycle.

The study was designed as a phase II Simon’s optimal two-stage design. The sample size for the first stage was 23 patients. If at least 12 patients reached the primary endpoint of DCR at eight weeks, the study would continue to enrol 14 more patients, totalising a sample of 37 patients. Otherwise, the study would be stopped. Considering a 10% dropout rate, the planned enrolment was 41 patients. For these assumptions, an alpha error of 5% and a beta error of 20% were considered.

We used descriptive statistics to report the absolute and relative frequencies. Time-to-event variables were estimated by the Kaplan-Meier method. Efficacy and safety analyses utilised the intention to treat principle.

The trial was approved by our local IRB and registered as NCT01971034.

## Results

From July 2011 to January 2014, N = 20 patients signed the informed consent, were enrolled and evaluable for analysis. Eight were men (40%) and 12 women (60%) with a median age of 62. One patient was diabetic and five patients had their primary tumour resected (25%). Fourteen patients had only one prior line of chemotherapy based on gemcitabine and six patients had received two prior lines of treatment; in all cases, the second line therapy was based on fluoropyrimidine. Table 1 summarises the characteristics of study participants.

Six patients reached the primary end point and all of them had stable disease after eight weeks of treatment. One patient lost follow-up. Therefore, it was chosen to terminate the study in the first phase.

The median number of cycles per patient was 1.5 (range 0.5–7). Eight patients (40%) had ≥ grade three adverse events, mostly gastrointestinal symptoms. (Table 2) Most grade 3 events were possibly related to paclitaxel, such as anaemia, neutropaenia and mucositis, or disease progression, such as bilirubin elevation and fatigue. Two patients had grade 3 diarrhoea and nausea which were associated with metformin. Six patients required dose-reduction of metformin to 850 mg b.i.d. because of diarrhoea and dyspepsia, while only one had to reduce the dose of paclitaxel to 60mg/m^2^ because of febrile neutropaenia.

The median OS was 128 days (range 17–697) and the median PFS was 44 days (range 14–210).

The median time to disease progression among all patients was 44 days. Analysing only those who did not meet the primary end point, the median time to progression (TTP) was 31 days, while it was 158.5 days for the six patients who met the primary end point.

After three months, 63.2% of all patients had disease progression and after six months only two of the six patients who met the primary end point had disease progression.

The analysis of the six patients who met the primary end point showed that when they failed experimental treatment, the images showed increased previous lesions and no new lesions.

## Discussion

This single-arm Simon’s two-stage phase II trial evaluating the activity of metformin combined with paclitaxel as a second line treatment for advanced cancer of the pancreas was negative. It showed that it was futile to advance to the next level of patient enrolment because the minimum established DCR was not achieved.

Pancreatic cancer is well recognised for being an aggressive disease, with only 50% of patients failing first-line treatment being eligible for further treatment [14]. In 2013, nab-paclitaxel combined to gemcitabine was shown to be better than gemcitabine alone as first line treatment for patients with advanced PAC [15]. By the time this study was initiated, these results were not available yet and gemcitabine was our standard first line treatment. In regards to second line treatment, there is no consensus in the literature about the best chemotherapy regimen. Taxanes have been investigated as a second line treatment option. In a phase II trial, docetaxel in combination to capecitabine was tested for patients whose tumour had progressed while on gemcitabine-based chemotherapy. Partial response was observed in three of 31 (9.7%) patients, stable disease in seven (22.6%), and disease progression in 21 (67.6%). Median PFS was only 2.4 months and the median OS was 6.3 months [16]. Another small phase II trial evaluated nab-paclitaxel monotherapy in second line for patients who progressed while on gemcitabine. The six-month OS was 58% among 20 patients and the median OS was only 7.3 months; one patient had a partial response, and six (32%) had stable disease as their best response [17]. This data suggests that these are critical patients whose disease responds poorly to treatment, and this is what challenges trial designs and conduction.

Recently two randomised phase III trials have shown survival gain in the second-line setting of pancreatic cancer. One of them is the CONKO-003(2) study, which was a phase III trial that randomised patients to folinic acid and fluorouracil (5-FU) or an oxaliplatin, folinic acid, and 5-FU based regimen. They included 160 patients and the analysis showed that patients treated with oxaliplatin have a median OS of 5.9 months compared to 3.3 months in the control arm. The other trial used nab-irinotecan MM-368 that also showed improved survival when this drug was combined with 5FU [3]. However, given the overall poor outcomes of these patients, other treatment strategies have eagerly been tested.

The general poor outcome of these patients helps to explain the three-year recruitment period needed in this study. In our cancer centre, the population treated is mostly characterised by advanced disease at arrival besides low performance status. Consequently, only a fraction of patients receive second line treatment and are eligible to be included in a clinical trial.

Although the literature supports a preclinical logical explanation for metformin use as an antineoplastic drug and large retrospective analysis have suggested that metformin was associated with improved outcomes when used in patients with diabetes and pancreatic cancer [11], our study did not confirm these hypothesis. Although our study enrolled only 20 patients, it is unlikely that increasing the number of patients would change our results. Actually to reinforce our negative results, a randomised phase II trial of gemcitabine with or without metformin in first line for pancreatic cancer did not find any benefit of adding metformin. [18].

The reasons why metformin was not effective in advanced pancreatic cancer are unknown. It is possible that the tumour microenvironment plays an important role in both drug penetration and in the survival of cancer cells. As demonstrated *in vitro* by Lonardo E *et al* in contrast to cancer stem cells that were ablated by metformin, the non-cancer stem cells entered cell cycle arrest when exposed to metformin, but were not eliminated [19]. In this same article, he evaluated primary cancer tissue xenograft models and showed that metformin reduced the tumour burden when allowed tissue penetration by a stroma-targeting smoothened inhibitor. Certainly, these aspects are not totally investigated or taken into account in preclinical models. Therefore, we would like to emphasise the importance of clinical trials aiming to reassure preclinical or observational data. Another explanation is the dose of metformin chosen to treat patients. The dose of 850 mg t.i.d. was selected because this is the maximum dose of metformin used by diabetic patients with reasonable tolerance. However, this dose was toxic to our patients and a considerable number of them had to have dose reduction and/or interruptions. But even among patients who received smaller doses of metformin and were more adherent to therapy, we could not document any efficacy in terms of tumour control.

Our trial discourages further investigation of metformin associated with paclitaxel for metastatic pancreas cancer. Nevertheless, we believe the literature supports subsequent investigation with other drug combinations and scenarios. Since metformin inhibits the mTOR/p3K pathway [6], it seems attractive to test this drug in tumours which harbour mTOR hyperactivation, such as neuroendocrine tumours (to be initiated by our group) and breast cancer (NCT01101438) [20].

## Conclusion

In conclusion, despite the encouraging pre-clinical evidence of antitumour activity of metformin in PAC, the primary endpoint of disease control rate was not met in our trial. Besides this, the treatment combination was poorly tolerated and should not be studied further in this setting.

Funding source: We did not receive any external funding for conducting the trial.

Ethics: The trial was approved by our local IRB and is registered as NCT01971034

## Figures and Tables

**Table 1.** Baseline characteristics.

All patients	N = 20 (100%)
Age (median, range)	62 years (44–77)
Sex (Male:Female)	8 (40): 12 (60)
Diagnosis of diabetes	1 (5)
Adjuvant chemotherapy	4 (20)
Primary tumour resected	5 (25)
Metastatic disease	20 (100)
Pre-treatment Ca19.9 ng/mL (median)	486
Baseline weight (kg)	58 (42–110)
ECOG: 0 1 2	2 (10) 10 (50) 8 (40)
Prior regimen First line gemcitabine only First line gemcitabine followed by second-line 5FU	14 (70) 6 (30)

**Table 2.** Proportion of patients who achieved grade 3/4 toxicities.

Adverse event	N (%)
Nausea	1 (5)
Vomiting	0
Mucositis	1 (5)
Diarrhoea	2 (10)
Neutropaenia	2 (10)
Anaemia	1 (10)
